# Rai14 is a novel interactor of Invariant chain that regulates macropinocytosis

**DOI:** 10.3389/fimmu.2023.1182180

**Published:** 2023-07-21

**Authors:** Natacha Lobos Patorniti, Khalisah Liyana Zulkefli, Martin E. McAdam, Pablo Vargas, Oddmund Bakke, Cinzia Progida

**Affiliations:** ^1^ Department of Biosciences, University of Oslo, Oslo, Norway; ^2^ Inserm U1151, Institut Necker Enfants Malades, Paris, France

**Keywords:** macropinocytosis, cell migration, cytoskeleton, invariant chain, antigen presenting cell (APC), Rai14, myosin II

## Abstract

Invariant chain (Ii, CD74) is a type II transmembrane glycoprotein that acts as a chaperone and facilitates the folding and transport of MHC II chains. By assisting the assembly and subcellular targeting of MHC II complexes, Ii has a wide impact on the functions of antigen-presenting cells such as antigen processing, endocytic maturation, signal transduction, cell migration, and macropinocytosis. Ii is a multifunctional molecule that can alter endocytic traffic and has several interacting molecules. To understand more about Ii’s function and to identify further Ii interactors, a yeast two-hybrid screening was performed. Retinoic Acid-Induced 14 (Rai14) was detected as a putative interaction partner, and the interaction was confirmed by co-immunoprecipitation. Rai14 is a poorly characterized protein, which is believed to have a role in actin cytoskeleton and membrane remodeling. In line with this, we found that Rai14 localizes to membrane ruffles, where it forms macropinosomes. Depletion of Rai14 in antigen-presenting cells delays MHC II internalization, affecting macropinocytic activity. Intriguingly, we demonstrated that, similar to Ii, Rai14 is a positive regulator of macropinocytosis and a negative regulator of cell migration, two antagonistic processes in antigen-presenting cells. This antagonism is known to depend on the interaction between myosin II and Ii. Here, we show that Rai14 also binds to myosin II, suggesting that Ii, myosin II, and Rai14 work together to coordinate macropinocytosis and cell motility.

## Introduction

Professional antigen-presenting cells (APCs) such as dendritic cells (DCs) and B-lymphocytes constitutively express Major Histocompatibility Class II molecules (MHC II), which consist of αβ heterodimers associated with self or antigenic peptides situated in their peptide-binding grooves (1). By processing antigens and presenting them for recognition to lymphocytes *via* MHC II, APCs are crucial for triggering an immune response. Invariant chain (Ii, CD74) is a type II transmembrane protein that self-associates into trimers and provides a scaffold for the assembly of MHC II heterodimers. Ii interacts with the peptide-binding groove of MHC II to prevent the premature binding of endogenous peptides and chaperones new MHC II molecules to endosomes for the loading of antigenic peptides (2, 3). Due to the presence of two leucine-based sorting motifs in its N-terminal cytosolic tail, Ii recruits adaptor proteins-1 and -2, which mediate the transport of new MHC II-Ii complexes to antigen-loading compartments from the *trans*-Golgi network, mainly *via* the cell surface (4, 5). In the endosomes, Ii is rapidly processed at the luminal side, leaving the Ii’s CLIP domain in the MHC II peptide-binding groove, which is thereafter replaced with antigenic peptides (3).

In the last couple of decades, it has become clear that in addition to assisting the assembly and transport of MHC II, Ii regulates multiple processes in APCs. For example, Ii mediates endosome fusion and delays endosome maturation (2, 6). Furthermore, by interacting with the actin motor myosin II, Ii regulates the macropinocytic and migratory ability of DCs in an antagonistic manner (7, 8). The antagonism between fast cell migration and antigen uptake depends on the localization of myosin II mediated by Ii (8). When myosin II is recruited to the cell front by Ii, the macropinocytic activity increases, reducing the migration speed. On the other hand, myosin II localization at the cell rear is necessary for fast migration (7, 8).

While the role of Ii in chaperoning MHC II is well established, the mechanisms regulating the other functions mediated by Ii are poorly understood. In this study, we search for novel interactors of Ii to shed light on these processes. For this, a yeast two-hybrid screen was performed using full-length Ii as bait, and Retinoic acid-induced 14 (Rai14) was one of the strongest positive candidates identified. Co-immunoprecipitation experiments further confirmed this interaction.

Rai14, also known as novel retinal pigment epithelial cell gene (NORPEG) and ankycorbin, is a member of a superfamily of ankyrin repeat proteins, termed N-Ank (9). The N-Ank protein superfamily includes proteins containing a set of ankyrin repeats and an N-terminal amphipathic helix that allows membrane interactions by insertion, senses membrane curvatures, and modulates membrane topologies. Their common function is membrane binding and shaping by combining electrostatic interactions of curvature-sensing ankyrin repeats and electrostatic and salt-insensitive hydrophobic interactions mediated by amphipathic helix insertion into one membrane leaflet (9). Rai14 is a good example of an N-Ank protein as it binds to membranes *via* both hydrophobic and electrostatic interactions. Indeed, it has been shown that Rai14 has a direct role in shaping membranes (9). The reported intracellular localization of Rai14 suggests that it also associates with the cortical actin cytoskeleton, F-actin stress fibers, and cell-cell adhesions sites (10–12). Furthermore, it has been proposed that Rai14 is a cytoskeleton-associated protein linked to actin function and organization (10, 11, 13, 14). Nevertheless, the function of Rai14 is still poorly characterized.

In this work, we report the previously undescribed role of Rai14 in macropinocytosis and cell migration. We show that Rai14 localizes to membrane ruffles and nascent macropinosomes. Furthermore, Rai14 silencing decreases the macropinocytosis in both MelJuSo cells and bone marrow-derived dendritic cells (BMDCs), delaying the internalization of MHC II. Knockdown of Rai14, similar to the depletion of Ii, increases the migratory ability of BMDCs. Ii is known to regulate the macropinocytic and migratory ability of DCs in an antagonistic manner by interacting with myosin II (7, 8). By showing that Rai14 interacts not only with Ii but also with myosin II, our study suggests that Rai14, myosin II, and Ii work in a complex to coordinate antigen uptake and migration in APCs.

## Results

### Rai14 is a novel interactor of Invariant chain

**Figure 1:**
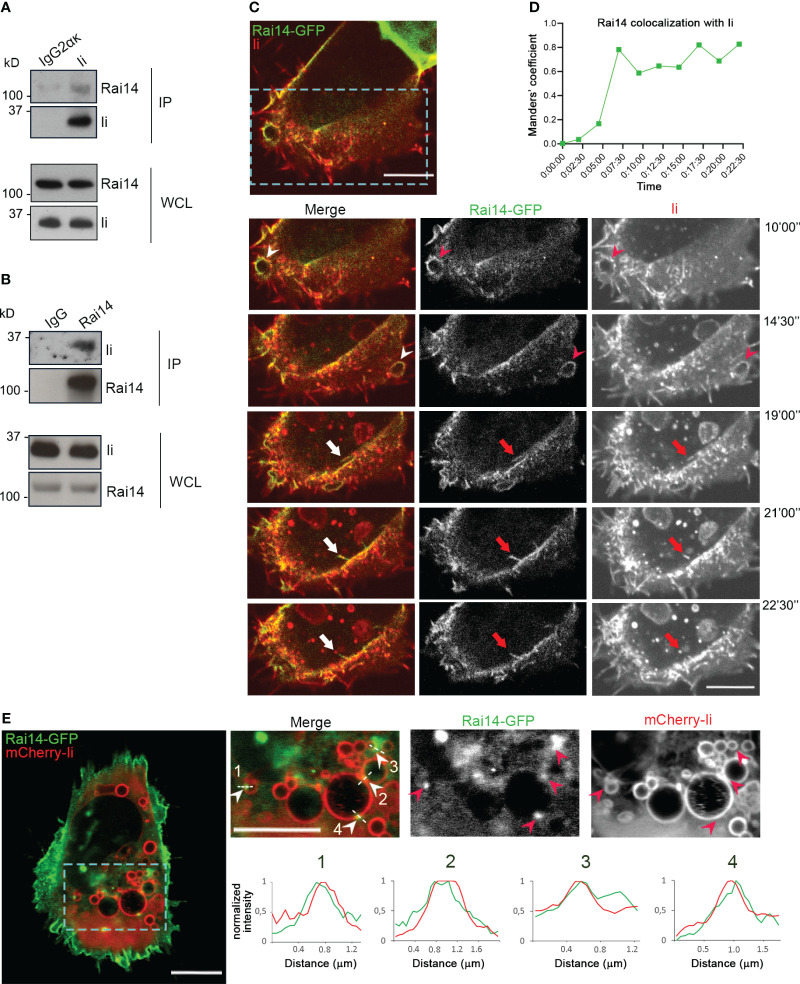
Ii interacts with Rai14. **(A)** Lysates from MelJuSo cells were subjected to immunoprecipitation with an antibody against Ii (MB741) or an isotype control (IgG2ακ). Immunoprecipitated (IP) samples and whole cell lysates (WCL) were analyzed by western blot using antibodies against Ii and Rai14. **(B)** MelJuSo cells were subjected to immunoprecipitation with an antibody against Rai14 or an isotype control (IgG). Immunoprecipitated (IP) samples and whole cell lysates (WCL) were analyzed by western blot using antibodies against Ii and Rai14. **(C)** An antibody targeting Ii (M-B741) conjugated with Alexa fluor 555 was added to MelJuSo cells co-transfected with Ii p33 and Rai14-GFP before live cell imaging. Arrowheads in the magnifications of the boxed area point to membrane ruffles positive for both Ii and Rai14, while the arrows point to a Rai14 and Ii-positive membrane tubule from where an Ii-positive vesicle pinches off. Scale bars, 10 μm. **(D)** Rai14 colocalization with Ii over time for **(C)** was quantified using Image J as Mander’s colocalization coefficients. **(E)** MelJuSo cells co-transfected with Iip33, mCherry-Iip33, and Rai14-GFP. Arrowheads in the magnifications of the boxed area point to Rai14 domains present on Ii-positive endosomes. Scale bars, 10 μm. The graphs show the normalized fluorescence intensity profiles relative to Rai14 (green) and Ii (red) along the lines illustrated in the insets.

To identify novel interaction partners of Invariant chain (Ii), a yeast two-hybrid screen was performed using Ii p33, the most abundant Ii isoform (15), as bait to screen a human placenta cDNA library. From this screen, Retinoic acid-induced 14 (Rai14) was identified as a positive hit (**Supplementary Table 1**). The human melanoma cell line MelJuSo is a good model for antigen-presenting cells (16) and, therefore, we used these cells to confirm the interaction of Ii with Rai14 by co-immunoprecipitation experiments. Rai14 was successfully pulled down by the endogenous Ii and not by a mouse IgG2α κ isotype control, indicating that Rai14 specifically binds to Ii (**Figure 1A**). To further confirm the interaction between Ii and Rai14, we also performed immunoprecipitation by pulling down Rai14 with an anti-Rai14 antibody. Also, in this case, endogenous Ii was co-immunoprecipitated by Rai14 and not by the IgG isotype control (**Figure 1B**). Taken together, these results confirm the interaction between Rai14 and Ii identified in the yeast two-hybrid screen.

To next elucidate whether Ii and Rai14 are present in the same cellular compartments, MelJuSo cells were co-transfected with Ii p33 and Rai14-GFP, and the uptake of antibodies targeting Ii labeled with Alexa Fluor 555 was analyzed using live cell imaging. Rai14-GFP was mainly localized at the plasma membrane and membrane ruffles, which also became internalized into vesicles (**Supplementary Figure 1A**; **Video 1**). Upon addition of the fluorescently labeled antibody targeting Ii, we detected Ii together with Rai14 on membrane ruffles, nascent vesicles, and forming macropinosomes. It was also possible to observe that Rai14 and Ii colocalized at very early stages of endocytosis until an Ii-positive vesicle pinched off (**Figures 1C, D**; **Video 2**). At steady state, in cells expressing mCherry-Ii, Rai14-GFP could additionally be detected in domains present on Ii-positive endosomes (**Figures 1D, E**).

### Rai14 depletion retains MHC II at the plasma membrane

**Figure 2:**
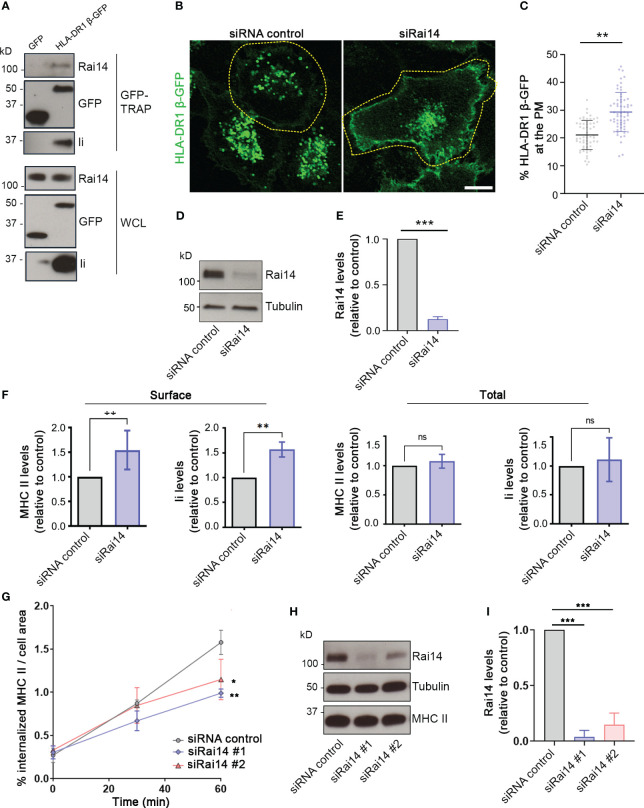
Rai14 is required for proper MHC II internalization. **(A)** MelJuSo cells stably expressing HLA-DR1 β-GFP or transfected with GFP were lysed and immunoprecipitated with GFP magnetic agarose beads. Whole-cell lysates (WCL) and immunoprecipitates (GFP-TRAP) were subjected to Western blot analysis using the indicated antibodies. **(B)** HLA-DR1 β-GFP MelJuSo cells were transfected with a siRNA control and a siRNA against Rai14 (siRai14) and imaged with a confocal laser scanning microscope. Scale bar: 10 μm **(C)** Quantification of the percentage of HLA-DR1 β-GFP present at the plasma membrane over the total in cells transfected with siRNA control or siRai14. The values represent the mean ± s.d. from three independent experiments. **P < 0.01 (two-tailed unpaired Student’s t-test). **(D)** Lysates from HLA-DR1 β-GFP MelJuSo transfected with siRNA control and siRai14 were analyzed by western blot using antibodies against Rai14 and tubulin as loading control. **(E)** Quantification of the endogenous Rai14 levels based on the intensity of the western blot bands was performed using FiJi. The data represent the mean ± s.d. normalized to the intensity of the tubulin and relative to the control, from three independent experiments. ***P < 0.001 (two-tailed unpaired Student’s *t*-test). **(F)** FACS analysis of the surface and total expression levels of HLA-DR and Ii for Rai14 siRNA-treated cells relative to control siRNA-treated cells. Surface levels were measured in intact cells (left panels), and total levels were measured in saponin-permeabilized cells (right panels) as mean fluorescence intensity. The data represent the mean ± s.d. from three independent experiments. **P < 0.01; ns, not significant (two-tailed unpaired Student’s *t*-test). **(G)** MelJuSo cells transfected with siRai14#1, siRai14#2, or siRNA control were incubated with an antibody against MHC II conjugated to Alexa Fluor 647 for 45 minutes and fixed at 0, 30, and 60 minutes after the treatment. Quantification of MHC II internalization measured as a percentage of the area occupied by MHC II-positive endosomes over the total cell area. Data represent the mean ± s.d. from three independent experiments (n > 45 cells per condition). *P < 0.05, **P < 0.01, for t = 60 min (two-tailed unpaired Student’s *t*-test). **(H)** Lysates from MelJuSo transfected with siRNA control, siRai14#1, or siRai14#2, were analyzed by western blot using antibodies against Rai14, MHC II, and tubulin as loading control. **(I)** Quantification of the endogenous Rai14 levels based on the intensity of the western blot bands was performed using FiJi. The data represent the mean ± s.d. normalized to the intensity of the tubulin and relative to the control, from three independent experiments. ***P < 0.001 (two-tailed unpaired Student’s *t*-test).

Having established that Rai14 interacts with Ii, we next investigated whether it also interacts with MHC II, as Ii forms a complex with nascent MHC II molecules and targets them to endocytic compartments. For this, lysate from MelJuSo cells stably expressing GFP-tagged MHC II (HLA-DR1 β-GFP) was subjected to immunoprecipitation using the GFP TRAP system. Cells expressing HLA-DR1 β-GFP (but not cells expressing GFP) were able to co-immunoprecipitate both endogenous Ii and Rai14 (**Figure 2A**), confirming that Rai14 interacts with Ii in a complex with MHC II.

Next, taking advantage of MelJuSo cells stably expressing HLA-DR1 β-GFP, we investigated whether Rai14 depletion has an effect on MHC II localization. In cells transfected with a siRNA control, HLA-DR1 β-GFP was present mostly on endosomes; however, the presence of HLA-DR1 β-GFP at the plasma membrane increased 1.5-fold in cells knocked down for Rai14 (**Figures 2B–E**). This suggests that Rai14 may be required for the internalization of the MHC II-Ii complex. Flow cytometry analysis indeed confirmed that the surface levels of both endogenous MHC II and Ii increased 1.5-fold in cells silenced for Rai14 compared to control cells, while the total levels remain unaffected (**Figure 2F**; **Supplementary Figure 2A**).

To confirm that the increased MHC II levels at the plasma membrane are due to a defect in MHC II internalization when Rai14 is knocked down, we then analyzed the uptake of MHC II in MelJuSo cells silenced for Rai14, using two different siRNAs targeting Rai14. MelJuSo cells were incubated with an antibody against MHC II conjugated to Alexa Fluor 647 and fixed at different time points (0, 30, and 60 minutes, **Supplementary Figure 2B**). Rai14 knocked-down cells internalized less MHC II in endosomes than the control cells, with a decrease of almost 40% of MHC II in endosomes 60 minutes after the uptake (**Figures 2G–I**). Altogether, these results indicate that Rai14 is required for MHC II internalization.

### Silencing of Rai14 inhibits macropinocytosis

**Figure 3:**
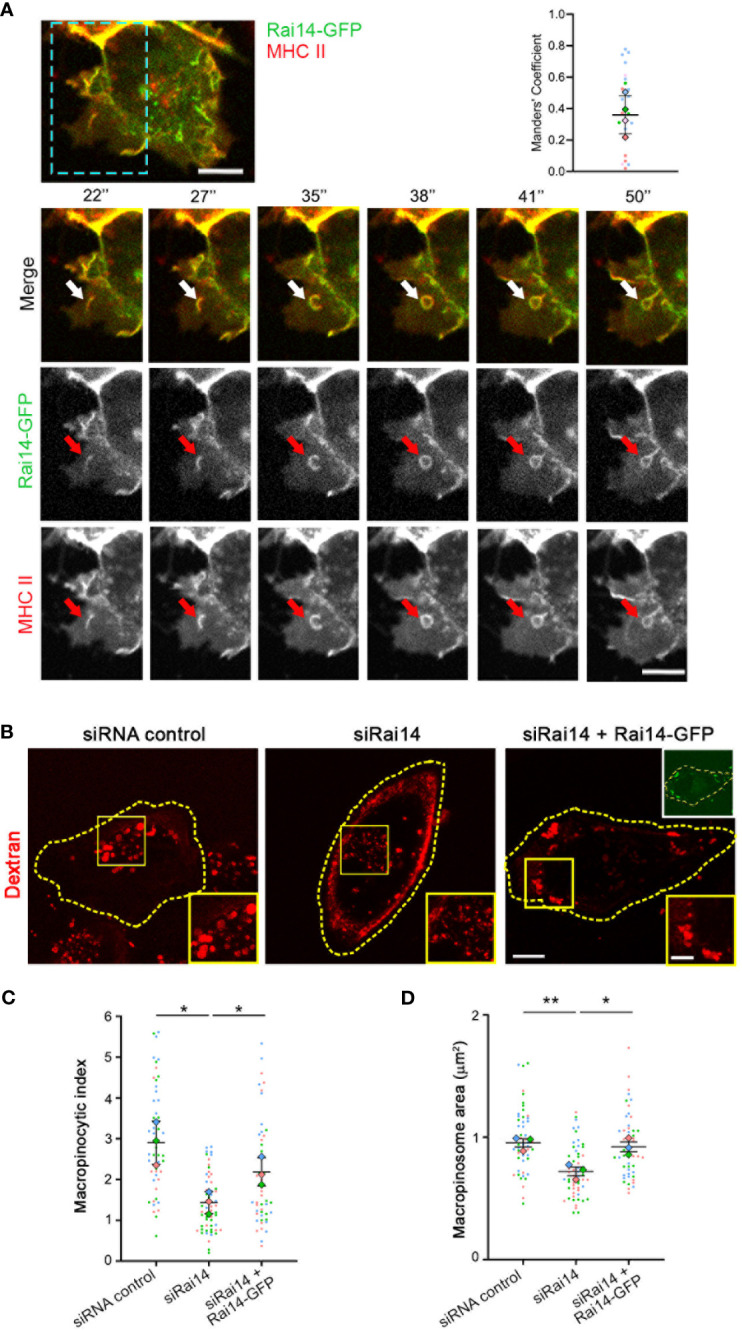
Rai14 and MHC II co-localize to membrane ruffles and forming macropinosomes. **(A)** Time-lapse video microscopy of MelJuSo cells transfected with Rai14-GFP. An antibody targeting HLA-DR (L243) labeled with Alexa 647 was added to the culture medium immediately before imaging. The arrows point to Rai14 and MHC II-positive membrane ruffle that close into a macropinosome. Scale bar = 10 µm. The graph to the right shows the colocalization analysis between Rai14 and MHC II using the JACoP plugin from ImageJ using Manders’ correlation coefficient. The data represent the mean ± s.d. from four independent experiments (n = 25). **(B)** MelJuSo cells treated with siRNA control, siRai14, or siRai14 and transfected with Rai14-GFP were incubated with 70 kDa Dextran Alexa Fluor 555 for 30 minutes. The images represent a maximum intensity projection of z stacks. The insets show magnifications of the boxed regions. Scale bar: 10 μm; inset: 5 μm. **(C)** Quantification of the macropinocytic index. The data represent the mean ± s.d. from three independent experiments (n = 60 cells per condition). *P < 0.05 (two-tailed unpaired Student’s *t*-test). **(D)** Quantification of the area of the macropinosomes. The data represent the mean ± s.d. from three independent experiments (n = 60 cells per condition). **P < 0.01; *P < 0.05 (two-tailed unpaired Student’s *t*-test).

To assess how Rai14 is involved in MHC II internalization, we analyzed the uptake of fluorescently labeled antibodies targeting MHC II in MelJuSo cells transfected with Rai14-GFP by live cell imaging. Intriguingly, Rai14-GFP and MHC II colocalized on membrane ruffles, and these ruffles lead to the formation of macropinosomes (**Figure 3A**; **Video 3**). Therefore, we next verified whether the inhibition of MHC II internalization upon Rai14 depletion was due to a defect in macropinocytosis. HLA-DR1 β-GFP MelJuSo cells transfected with siRNA against Rai14 or with siRNA control were incubated with fluorescently labeled dextran to visualize macropinosomes. Live cell imaging analysis revealed that the macropinocytic index, which corresponds to the percentage of cell area occupied by dextran-positive macropinosomes within a given time frame of dextran uptake (17), decreased by half in cells silenced for Rai14, and that this was a consequence of smaller macropinosome area (**Figures 3B–D**). The reintroduction of Rai14 in cells silenced for this protein rescued the macropinocytosis defect, further validating the specificity of Rai14 siRNA (**Figures 3B–D**).

**Figure 4:**
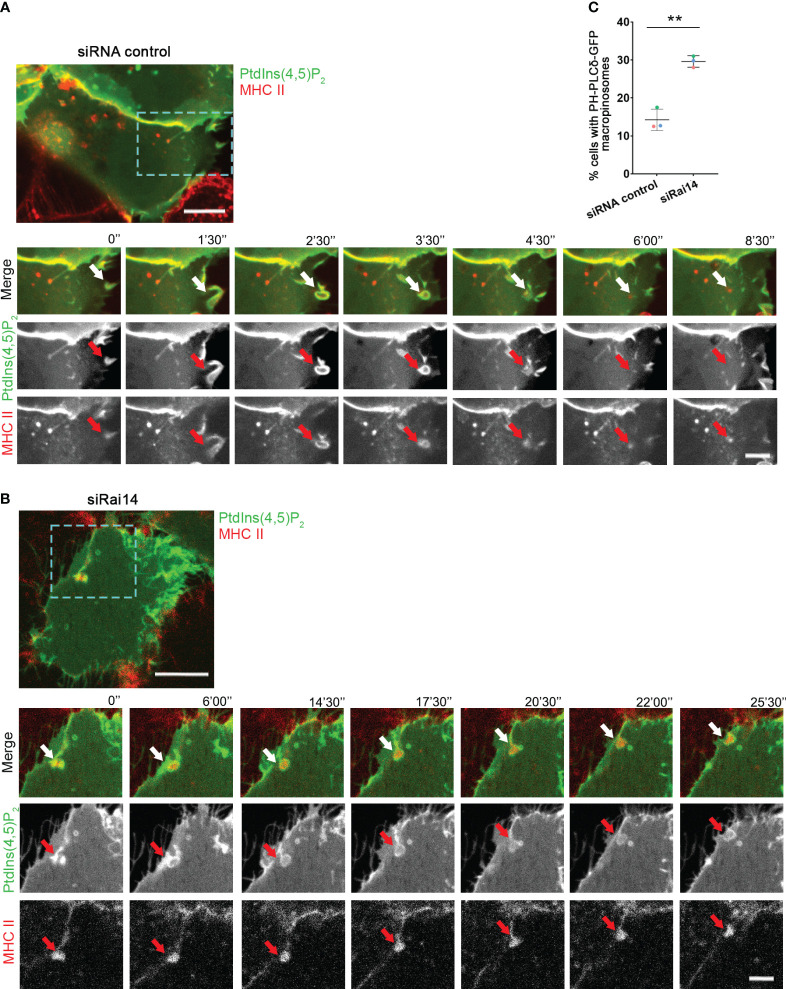
PtdIns(4,5)P_2_ persists at the macropinocytic cup in cells depleted for Rai14. **(A)** Time-lapse video microscopy of MelJuSo cells treated with siRNA control and transfected with PH-PLCδ-GFP to visualize PtdIns(4,5)P_2_. An antibody targeting HLA-DR (L243) labeled with Alexa 647 was added to the culture medium immediately before imaging. The arrows point to PtdIns(4,5)P_2_ and MHC II-positive membrane ruffle that closes into a macropinosome and loses PtdIns(4,5)P_2_. Scale bar = 10 µm; inset: 5 μm. **(B)** Time-lapse video microscopy of MelJuSo cells treated with siRai14 and transfected with PH-PLCδ-GFP to visualize PtdIns(4,5)P_2_. An antibody targeting HLA-DR (L243) labeled with Alexa 647 was added to the culture medium immediately before imaging. The arrows point to PtdIns(4,5)P_2_ and MHC II-positive membrane ruffle that retains PtdIns(4,5)P_2_ at the macropinocytic cup. Scale bar = 10 µm; inset: 5 μm. **(C)** Quantification of the percentage of cells with retaining PtdIns(4,5)P_2_ at the membrane of forming macropinosomes. The data represent the mean ± s.d. from three independent experiments (n ≥ 32 cells per condition). **P < 0.01 (two-tailed unpaired Student’s t-test).

Having assessed that Rai14 has a role in macropinocytosis, we next investigated in which part of this process it is specifically involved. Macropinosome formation involves loss of PtdIns(4,5)P_2_, which is present at the plasma membrane, from the macropinosome membrane as it internalized (18–20). We, therefore, took advantage of the PtdIns(4,5)P_2_ biosensor PH-PLCδ-GFP to visualize the membrane of nascent macropinosomes and macropinocytic cups. As expected, in the majority of the cells transfected with siRNA control, PtdIns(4,5)P_2_ was quickly depleted upon macropinosome formation (**Figures 4A, C**; **Video 4**). Intriguingly, in cells silenced for Rai14, the percentage of cells retaining PtdIns(4,5)P_2_ at the membrane of nascent macropinosomes dramatically increased (**Figures 4B, C**; **Video 5**), suggesting that Rai14 is required for macropinosome closure.

**Figure 5:**
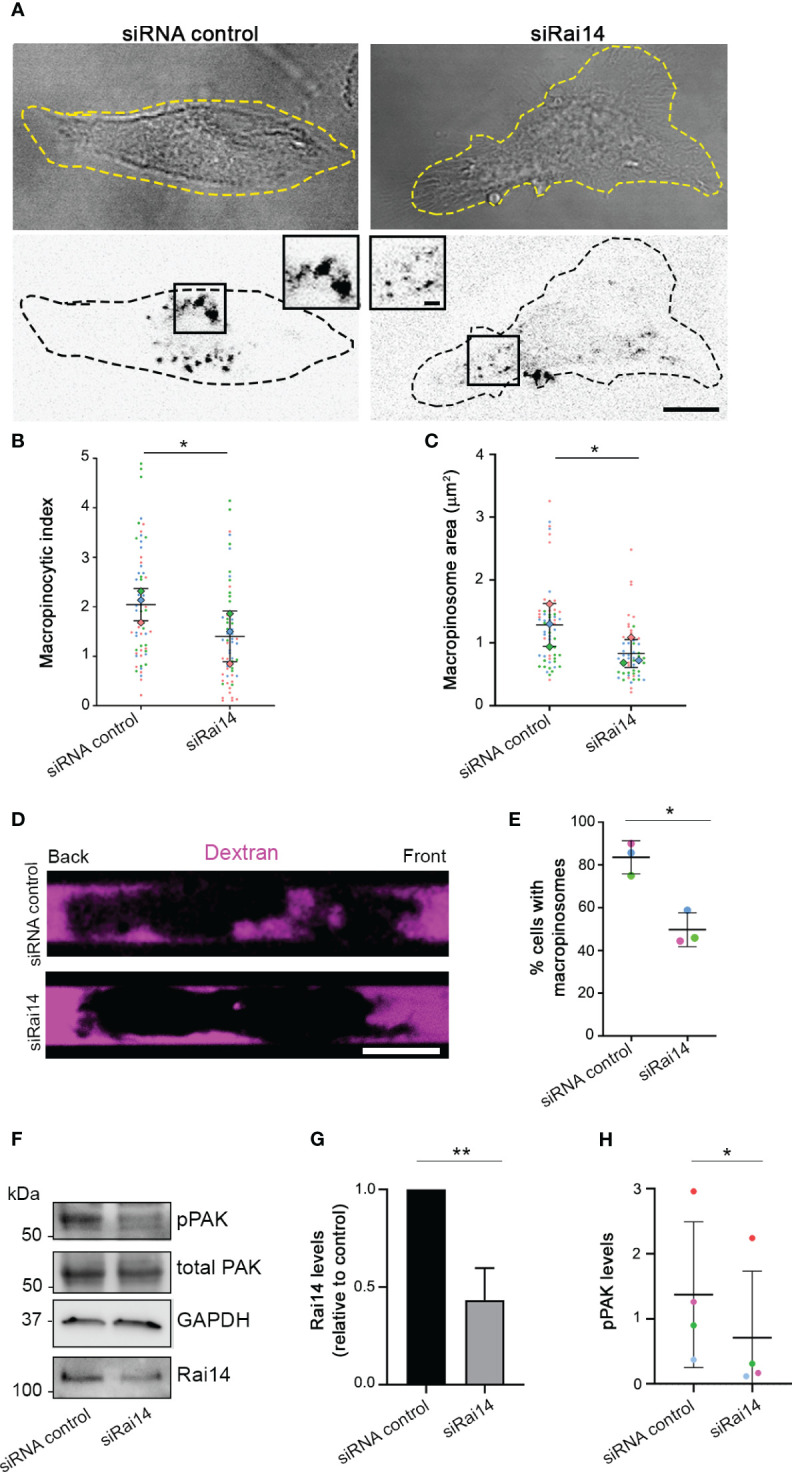
Rai14 silencing reduces macropinocytosis in BMDCs. **(A)** BMDCs were transfected with siRNA control and siRNA Rai14 and incubated with 70 kDa Dextran Alexa Fluor 555 for 15 minutes. The dextran channel has been inverted to improve visualization. Scale bar: 10 μm; inset: 2 μm. **(B)** Quantification of the macropinocytic index. The data represent the mean ± s.d. from three independent experiments (n = 60 cells per condition). *P < 0.05 (two-tailed paired Student’s *t*-test). **(C)** Quantification of the area of the macropinosomes. The data represent the mean ± s.d. from three independent experiments (n = 60 cells per condition). *P < 0.05 (two-tailed paired Student’s *t*-test). **(D)** BMDCs treated with siRNA control or siRai14 were loaded into 5×8 µm micro-fabricated channels. After 16 h, the channels were filled with 10 kDa Alexa Fluor 555-conjugated dextran (magenta), and the cells were imaged 50 min later. Scale bar: 10 µm. **(E)** Quantification of the percentage of cells with internalized dextran. The graph represents the mean ± s.d. from three independent experiments (n ≥ 40 cells per condition). *P < 0.05 (two-tailed paired Student’s *t*-test). **(F)** Lysates from BMDCs treated with siRNA control or siRai14 were analyzed by western blot using antibodies against Rai14, phosphorylated PAK, and total PAK. GAPDH antibody was used as loading control. **(G)** Quantification of Rai14 levels normalized to GAPDH and represented relative to control. Data represent the mean ± s.d. from five independent experiments. **P < 0.01 (two-tailed paired Student’s *t*-test). **(H)** Quantification of the levels of phosphorylated PAK in BMDCs normalized to total PAK and tubulin. Data represent the mean ± s.d. from four independent experiments *P < 0.05 (two-tailed paired Student’s *t*-test).

To confirm the role of Rai14 in macropinocytosis, we verified whether Rai14 depletion affects this process also in DCs, which are cells with high constitutive macropinocytic activity (21). In primary bone marrow-derived DCs (BMDCs) silenced for Rai14, there was a 30% decrease in the macropinocytic index (**Figures 5A, B**). Similarly, the macropinosome area decreased by approximately 30% in cells knocked down for Rai14 (**Figure 5C**). To further define the impact of Rai14 on macropinocytosis, we analyzed dextran uptake in BMDCs confined in microchannels. Microchannels are a useful tool for the study of cell migration and macropinocytosis in BMDCs due to the cell confinement forcing them to adopt a similar polarized elongated shape, allowing differentiation between front and back (8, 22, 23). Analysis of BMDCs in microchannels revealed an almost 40% decrease in the number of cells containing macropinosomes upon Rai14 depletion (**Figures 5D, E**).

We next investigated whether the decrease in macropinocytosis upon Rai14 silencing is reflected by a reduction of p21-activated kinase (PAK) activity. PAK is a serine/threonine kinase that, upon activation, is auto-phosphorylated, promoting actin cytoskeleton remodeling and macropinocytosis (24, 25). Interestingly, quantification of the levels of PAK phosphorylation revealed a decrease of almost 50% in BMDCs silenced for Rai14 compared to control cells (**Figures 5F–H**). Altogether, these results indicate that Rai14 is required for macropinocytosis.

### Rai14 negatively regulates BMDC migration

**Figure 6:**
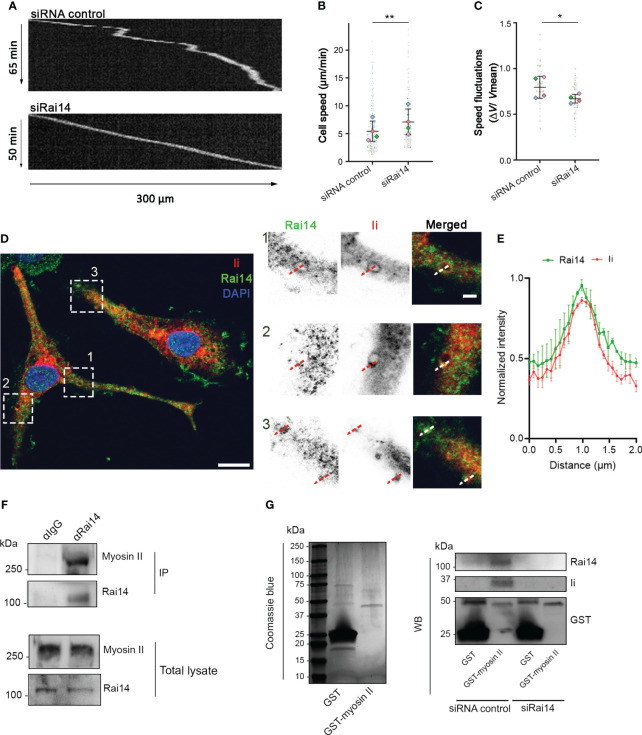
Rai14 interacts with myosin II and affects migration of BMDCs. **(A)** BMDCs transfected with either siRNA control or siRai14 were loaded in 5×8 μm micro-fabricated channels and imaged for 20 h in an epifluorescence Olympus microscope, using a 10X objective and acquiring one transmission phase image every minute. Representative kymographs are shown. **(B)** Quantification of the mean ± s.d cell speed (µm/min) from four independent experiments (n > 150 cells per condition). **P<0.01 (two-tailed paired Student’s *t*-test). **(C)** Quantification of mean ± s.d. speed fluctuations [calculated as s.d./mean instantaneous speed (7, 8, 23) from four independent experiments (n > 150 cells per condition). *P<0.01 (two-tailed paired Student’s *t*-test). **(D)** BMDCs were fixed and stained with antibodies against Rai14 (green) and Ii (red). Nuclei were stained with DAPI (blue). The image contrast and brightness have been increased to allow for easier visualization of the staining. The insets show magnifications of the boxed regions. Scale bars: 10 μm; inset: 2 μm. **(E)** Normalized fluorescent intensity profile for Rai14 and Ii along the lines as illustrated in the insets in D). Data represent the mean ± s.d. from two independent experiments. n = 78 vesicles from 37 cells. **(F)** DC lysates were subjected to immunoprecipitation with an antibody against Rai14 or an isotype control (IgG). Total lysate and immunoprecipitates (IP) were subjected to western blot analysis using antibodies specific to myosin II and Rai14. **(G)** Left panel: Coomassie Blue staining of bacterially expressed GST and GST–myosin II heavy chain tail purified using glutathione resin. Right panel: purified GST or GST–myosin II heavy chain tail were incubated with lysates from MelJuSo cells transfected with siRNA control or siRai14. Samples were subjected to affinity chromatography followed by western blot analysis using antibodies specific to GST, Rai14, and Ii.

Immature DCs are characterized by high macropinocytic activity as they sample the environment by engulfing large amounts of extracellular material to search for antigens. High macropinocytic activity corresponds to phases of slow motion due to the recruitment of myosin II at the DC front (8). As our results indicate that Rai14 regulates macropinocytosis, and macropinocytosis and cell migration are antagonistic processes, we investigated whether Rai14 also influences cell migration. BMDCs transfected with control siRNA or siRNA targeting Rai14 were loaded into microchannels, as DCs migrate faster in confined environments (26, 27), and imaged overnight. The analysis revealed that BMDCs silenced for Rai14 move faster (mean speed 7.1 µm/min) than control BMDCs (mean speed 5.4 µm/min) (**Figures 6A, B**). In addition, upon depletion of Rai14, BMDCs show fewer local speed variations compared to control cells, indicating that the cells knocked down for Rai14 change direction less frequently (**Figures 6A–C**).

Intriguingly, it is known that DCs depleted for Ii display a similar behavior, and this occurs because Ii interacts with myosin II (7). By interacting with myosin II, Ii recruits this motor protein at the front of migrating DCs to promote macropinocytosis and decrease migration (8). We, therefore, wondered whether Rai14 is the missing link between Ii and myosin II to couple antigen uptake and migratory ability. To verify this hypothesis, we first investigated whether Rai14 depletion, similar to Ii knockout (8), prevents myosin II recruitment at the leading edge of migrating BMDCs. Silencing of Rai14 indeed decreased the amount of myosin II at the front of BMDCs migrating into microfabricated channels, as revealed by density maps of the mean myosin II distribution (**Supplementary Figure 3**). We next evaluated the endogenous localization of Rai14 and Ii in BMDCs. Our results show that, in line with our hypothesis, the two proteins colocalize on large, macropinosome-like vesicles (**Figures 6D, E**). Next, we assessed whether Rai14 also interacts with myosin II. Co-immunoprecipitation experiments revealed that Rai14 is indeed able to bind myosin II (**Figure 6F**). In addition, while purified GST-tagged myosin II heavy chain tail specifically precipitates both Ii and Rai14 from total MelJuSo cell extracts, it is unable to pull down Ii from cells silenced for Rai14 (**Figure 6G**), suggesting that Rai14 bridges Ii to myosin II.

Altogether, our results indicate that Rai14, Ii, and myosin II work together to coordinate antigen uptake, intracellular membrane traffic, and, hence, cell migration in antigen-presenting cells.

## Discussion

Ii is well known for its crucial role in antigen presentation by mediating folding and subcellular trafficking of MHC II from the ER to the plasma membrane and to the antigen loading compartment (28–31). However, Ii is responsible for many other functions in antigen-presenting cells, including antigen processing (32), endosomal maturation (6, 33), signal transduction (30), cell migration, and macropinocytosis (7, 8). These functions are made possible through interactions of Ii with other proteins (6, 7, 30), but the underlying molecular mechanisms are less characterized.

Efficient antigen uptake by macropinocytosis requires myosin II. Myosin II is also fundamental for another important function of DCs: cell migration. There is indeed antagonism between fast cell migration and antigen uptake that relies on the regulation of myosin II localization by Ii (8). Myosin II at the cell rear is necessary for DC migration. However, when myosin II is recruited by Ii to the cell front, the cells reduce their migration speed and increase their macropinocytic activity (7, 8). Nevertheless, how Ii coordinates macropinocytosis and cell migration by affecting myosin II localization is not well understood.

In this work, we show that depletion of Rai14 reduces macropinocytosis and promotes DC migration. Intriguingly, these results are similar to those reported for BMDCs lacking Ii or myosin II. Indeed, similarly to BMDCs silenced for Rai14, myosin II or Ii knockout BMDCs display smaller macropinosomes (8). Furthermore, like Rai14-depleted cells, Ii-deficient DCs migrate faster, exhibit fewer speed fluctuations than wild-type cells (7), and fail to recruit myosin II at the cell front for macropinocytosis (8). As our data indicate that Rai14 interacts with myosin II, it is likely that it negatively regulates cell migration by directly recruiting myosin II at the front of migrating cells to promote macropinocytosis. In line with that, Rai14 depletion displaces myosin II from the leading edge of migrating cells, decreasing macropinocytosis and promoting cell motility. In addition, Rai14 silencing inhibits PAK activation, which, in agreement with previous reports, also contributes to myosin II displacement from the cell’s leading edge (34).

Our results, therefore, suggest that Rai14 is the possible link connecting Ii and myosin II for the coordination of macropinocytosis, intracellular membrane traffic, and cell migration in antigen-presenting cells. Further supporting this, we found that Rai14 interacts with both Ii and myosin II and that the lack of Rai14 prevents the Ii-myosin II association. In line with this, it has been previously described that Rai14 indirectly associates with the actin cytoskeleton by possible interactions with other actin-binding proteins (10, 11). Therefore, Rai14 is likely a scaffold protein that links Ii with myosin II and the actomyosin network.

Even if not much is known about Rai14 function, Rai14 is a scaffold protein that seems to be involved in actin polymerization (11, 12) and, more recently, it has been found to also regulate membrane shaping (9). The link between plasma membrane curvature and the cortical actin cytoskeleton is highly essential during endocytic mechanisms such as macropinocytosis, modulations of membrane tensions, and the shaping of entire cells (35). It is therefore possible that Rai14 acts as a link between membrane shaping and actin in macropinocytosis. In agreement with this, we observed Rai14-GFP at the plasma membrane, on membrane ruffles and closing macropinosomes in live cells. Rai14 is a member of the N-Ank protein family. The members of this family are characterized by an ankyrin repeat array and an N-terminal amphipathic helix to bind and shape membranes (9). Interestingly, another member of the N-Ank protein family, ANKHD1, induces fission on early endosomes (36). As we also observed Rai14 at the sites of vesicle scission from membrane tubules or enriched on endosomal domains (**Figures 1C, D**), it is tempting to speculate that Rai14 could also be involved in membrane fission.

Taken together, our results indicate that Rai14 is necessary for the antagonist coordination of macropinocytosis and cell migration in antigen-presenting cells. The interaction of Rai14 with both Ii and myosin II further suggests that this coordination is possible as Rai14 may act as a scaffold protein linking Ii to the actin motor.

## Materials and methods

### Cell culture

The human melanoma MelJuSo wt and HLA-DR1 β chain-GFP (HLA-DR1 β-GFP) stably transfected MeljuSo cells (16) were grown in Iscove’s Modified Dulbecco’s Medium (IMDM, Gibco) supplemented with 10% fetal calf serum (FCS), 2 mM L-glutamine, 100 U/ml penicillin, and 100 μg/ml streptomycin. Bone marrow-derived DCs (BMDCs) from 8–16-week-old male or female C57BL/6 mice (37) were used in this study. BMDCs were generated as previously described (23) and cultured in medium supplemented with 10% decomplemented FCS, 2 mM L-glutamine, 100 U/ml penicillin, 100 μg/ml streptomycin, 50 ng/ml granulocyte-macrophage colony-stimulating factor-containing supernatant obtained from transfected J558 cells, as previously described (7), and 14.3mM β-mercaptoethanol. MelJuSo and BMDCs cells were maintained in a 5% CO_2_ atmosphere at 37°C. All experiments were performed in accordance with relevant guidelines and regulations. The animals were bred under conventional conditions, regularly screened for common pathogens, and housed in compliance with guidelines set by the Experimental Animal Board under the Ministry of Agriculture of Norway. All experimental protocols involving animals were approved by the National Committee for Animal Experiments (Oslo, Norway).

### Antibodies, constructs, and reagents

The following antibodies were used for immunofluorescence (IF), immunoprecipitation (IP), and Western blot (WB) experiments:

anti-Rai14 (ab137118, abcam, IF 1:40, WB 1:500, IP 1:200), anti-alpha tubulin (T9026, Sigma-Aldrich, WB 1:10000), anti-human CD74 (MB741) (555538, BD biosciences, WB 1:500, IP 1:200), anti-mouse CD74 (In-1) (555317, BD biosciences, IF 1:50), isotype control anti-IgG2aK (555571, BD biosciences, IP 1:200), isotype control anti-IgG (550875, BD biosciences, IP 1:200), anti-GFP antibody (ab6556, abcam, WB 1:1000), anti-HLA-DR (L243) (555809, BD biosciences, IF 0.5 mg/μl), anti-HLA-DR (TAL-1B5) (ab20181, abcam, IF 1:200, WB 1:50), anti-PAK 1/2/3 (2604S, Cell Signaling Technology, WB 1:500), anti-phospho-PAK1 (Ser144)/PAK2 (Ser141) (2606S, Cell Signaling Technology, WB 1:200), anti-GAPDH (MAB374, chemicon, WB 1:3000), anti-non-muscle myosin IIA (ab24762, abcam, WB 1:2000). Anti-Ii (MB741) and anti-MHC II (L243) were labeled for live immunostaining or flow cytometry using a monoclonal antibody labelling kit (Molecular Probes, Eugene, Oregon, USA) according to the manufacturer’s protocol, with Alexa Fluor -555 or -647.

Secondary antibodies conjugated to Alexa Fluor ^®^ 488, Alexa Fluor ^®^ 555, or Alexa Fluor ^®^ 647 fluorophores (Invitrogen, 1:200) were used for IF; horse-radish peroxidase (HRP)-conjugated rabbit anti-mouse IgG and HRP-conjugated swine anti-rabbit IgG (GE Healthcare, Chicago, Illinois, USA, 1:5000) were used for WB.

pCMV6-AC-Rai14-GFP was purchased from OriGene Technologies, Inc., and pEGFP-C1 from BD Biosciences. pcDNA3 Ii p33, pcDNA3-mCherry-Iip33, and pGEX2T-myosin II heavy chain tail (amino acids 1795–1960) have been described before (38–40). PH-PLCδ-GFP was a kind gift from Tamas Balla (Eunice Kennedy Shriver National Institute of Child Health and Human Development, Bethesda, Maryland, USA). DAPI (D9542, Sigma-Aldrich) was used at 0.2 μg/ml, 10 kDa dextran Alexa Fluor^®^ 555 conjugated (Invitrogen) was used at 100 μg/ml, 70 kDa dextran Alexa Fluor^®^ 555 conjugated (Molecular Probes) was used at 100 μg/ml. Fibronectin (Sigma-Aldrich) was used at 20 μg/ml for the coating of coverslips and microchannel chips.

### RNA interference

For RNA interference (RNAi) in human cells, the following siRNA oligos were used;

Rai14 siRNA#1, sense sequence 5′- UCAUCUCCAUCUGUCUUAATT -3′ and antisense sequence 5′- UUAAGACAGAUGGAGAUGATT -3′.

Rai14 siRNA#2, sense sequence 5′- AUAUUCAGACUCUCUUGAATT -3′ and antisense sequence 5′- UUCAAGAGAGUCUGAAUAUTT -3′.

As negative control, we used the sense sequence 5′-ACUUCGAGCGUGCAUGGCUTT-3′ and antisense control 5′-AGCCAUGCACGCUCGAAGUTT-3′. The oligos were purchased from Eurofins MWG Operon.

For RNA interference (RNAi) in murine DCs, we used FlexiTube Rai14 siRNA, cat. no. SI01396318; and as negative control, the sense sequence 5′-UUCUCCGAACGUGUCACGUTT-3′ and antisense sequence 5′-ACGUGACACGUUCGGAGAATT-3′. The murine oligos were purchased from Qiagen.

### Transfection and RNA interference

MelJuSo cells were transiently transfected at a confluency of approximately 50%–70% using Lipofectamine 2000 (Life Technologies) according to the manufacturer’s protocol, for 24 hours. Transfection with siRNAs was performed using Lipofectamine RNAiMAX Transfection Reagent (Life Technologies) following the producer’s protocol for 72 hours.

BMDCs were collected on day 7 of differentiation and transfected using the Amaxa Biosystems Nucleofector II electroporator (Lonza). Cells were resuspended to 2-2.5 x 10^6^ cells per 100 µl of cold PBS 1X containing 1 µM siRNA. The cell suspension was transferred into an aluminum cuvette with a 2 mm gap size (Lonza) and electroporated using the program Y-001. Cells were incubated at 37°C for 30 minutes before washing steps and re-plating for later experiments on day 10.

### Co-immunoprecipitation

Dynabeads Protein G (ThermoFisher Scientific) or GFP-Trap_MA (Chromotek) were used for co-immunoprecipitation experiments according to the producers’ protocol. Briefly, antibodies were conjugated to the Dynabeads at room temperature with end-over-end rotation for 1 hour. Cells were lysed in RIPA buffer (150 mM NaCl, 50 mM Tris-HCl pH 7.8, 1% Sodium Deoxycholate, 0.1% Sodium dodecyl sulfate (SDS), 1.5% Triton X-100, and 1X Protease Inhibitor Cocktail (Roche Diagnostics)) and the precleared lysates were incubated for 1.5 hours at room temperature with magnetic beads coupled to the specific antibody.

Immunoprecipitation experiments using GFP-Trap magnetic beads were performed as previously described (41). Briefly, cells transfected with GFP-fusion proteins were lysed in lysis buffer (10 mM Tris-HCl pH 7.5, 150 mM NaCl, 0.5 mM EDTA, and 0.1% NP-40) and the lysates were incubated for 1 h at 4°C with magnetic beads coupled to anti-GFP. The immunoprecipitated samples and their respective total lysates were loaded on SDS-PAGE gels and analyzed by western blotting.

### Protein purification and pulldown experiments

GST-tagged proteins were expressed in Escherichia coli BL21 (DE3) (Agilent Technologies) upon induction with 0.5 mM IPTG for 4 h at 37°C. Following centrifugation, bacteria were resuspended in 64 mM Tris-HCl pH 8.5, 8 mM MgCl_2_, 20 mM β-mercaptoethanol, 0.30 mM PMSF, 0.8 ng lysozyme/gram pellet, and 10 μg/ml DNase and lysed by French press. After centrifugation at 48,000 × g for 1 h at 4°C, the GST-tagged proteins present in the soluble fractions were purified with Glutathione Sepharose 4B affinity chromatography resin (Cytiva). Bacterial soluble fractions were incubated with 50% resin in PBS 1X at room temperature with end-over-end rotation for 30 minutes. The resin was washed with cold PBS 1X and the GST-tagged proteins were eluted from the resin by using glutathione elution buffer (10 mM glutathione, 50 mM Tris-HCl, pH 8.0). Protein concentration and purity were measured with an ND-1000 Spectrophotometer (Thermo Scientific).

Pulldown experiments were performed using the Glutathione Sepharose 4B affinity chromatography resin (Cytiva). 10 μg of purified GST-tagged proteins were bound to the resin and incubated with 200 μl of precleared MelJuSo cell lysates for 30 min at 4°C. The resin was washed with cold PBS 1X and the GST-tagged proteins and their interactors were eluted from the resin by using a glutathione elution buffer (10 mM glutathione, 50 mM Tris-HCl, pH 8.0). Samples were analyzed using SDS-PAGE and immunoblotting.

### Western blotting

Cells were lysed for 15 min on ice followed by 10-min centrifugation at 4°C. The clarified supernatant was measured for protein concentration using the Bradford assay (Bio-Rad, UK). Cell lysates were resolved on an SDS-PAGE at 130 V for 1 hour and transferred onto a PVDF membrane (Millipore) at 150 mA for 3 hours at 4°C. The membrane was blocked and rehydrated with washing buffer, 0.1% Tween-20/in PBS 1X, and incubated with primary antibodies in 2% non-fat dry milk (BioRad) overnight at 4°C, followed by incubation with secondary HRP-conjugated antibodies for 1 hour at room temperature. ECL Prime Western Blotting Detection (GE Healthcare) was used to detect the chemiluminescent signals. Quantification of band intensity was done by densitometry analysis with Image Lab Software (Bio-Rad).

### Flow cytometry

MelJuSo cells were detached using trypsin-EDTA for 5 minutes and neutralized with complete IMDM. For cell surface staining, cells were incubated with either Alexa 647-conjugated L243 or Alexa 647-conjugated MB741 antibodies for 45 minutes on ice. Cells were then fixed with 3% PFA overnight. For total protein staining, cells were fixed and permeabilized with permeabilization buffer (0.1% saponin, 1% BSA in PBS 1X) and incubated with either Alexa 647-conjguated L243 or Alexa 647-conjugated MB741 for 45 minutes at room temperature. Samples were run through the BD Fortessa™ and results were analyzed using FlowJo software (Tree Star Inc, USA).

### Yeast two-hybrid screen

A yeast two-hybrid screen of a human cDNA library from placenta using human Ii p33 as bait was performed by Hybrigenics Services (Paris, France).

### Immunofluorescence

Cells grown on glass coverslips were fixed with 3% PFA for 20 minutes at room temperature. This was followed by a 10-minute quenching step with 50 mM NH_4_Cl. Cells were then permeabilized with 0.2% saponin (Sigma-Aldrich) in PBS for 10 min before being washed and incubated with primary antibodies for 20 minutes. This was followed by three washes with 0.2% saponin in PBS before the incubation with Alexa Fluor-conjugated secondary antibodies for 20 minutes. After another three washes, the cells were incubated with DAPI for 5 min at room temperature. Finally, cells were washed with PBS and distilled water and mounted with Mowiol (Sigma-Aldrich). Imaging was performed using an inverted Olympus FluoView 1000 IX81 confocal laser scanning microscope equipped with a 60× PlanApo NA 1.35 objective. For imaging of HLA-DR L243 Alexa Fluor 647 internalization in MelJuSo cells, a Zeiss LSM880 microscope equipped with a 63× oil Plan Apo NA 1 objective was used.

### Preparation of microchannels and speed quantification

Microchannels were prepared as previously described (7, 8, 42). Briefly, microfluidic devices were fabricated in polydimethylsiloxane (PDMS) using rapid prototyping and soft lithography. PDMS pieces and a glass-bottom FluoroDish (World Precision Instruments) were activated in a plasma cleaner (PDC-32G Harrick) for 30 s and were stuck together. They were then incubated with 20 mg/ml fibronectin for 20 minutes and washed with PBS 1X. BMDCs were incubated with DAPI for 45 minutes before being loaded and allowed to enter the microchannels. For velocity quantification, cells were imaged over 16 hours on an epifluorescence video-microscope Nikon TiE microscope equipped with a cooled CCD (charge-coupled device) camera (HQ2, Photometrics) with a 10× objective, taking one image every 1 minute. Kymograph extraction and velocity analysis were performed using ImageJ.

### Myosin II density map generation and analysis

10^6^ BMDCs were loaded in 5×8 μm microchannels coated with 20 μg/ml of fibronectin and allowed to migrate overnight at 37°C, 5% CO_2_. The cells were then fixed with 3% PFA for 20 min and washed with PBS. The PMDS structure on top of the channels was carefully removed and the cells were permeabilized with 0.25% saponin in PBS for 10 min and immunostained. The cells were imaged using an inverted Olympus Fluoview FV1000 confocal laser scanning microscope equipped with a 60× PlanApo NA 1.35 objective.

ImageJ was used to generate density maps as previously described (23). Briefly, images were cropped to contain single cells and resized to the average cell size. Next, the background was subtracted, and the intensities were normalized. Finally, the mean signal of every individual cell was projected to generate density maps and the physics look-up table (LUT) was applied to the image. Intensities were measured in rectangles corresponding to the front (20% of the cell) and the back (20% of the cell).

### Macropinocytosis assays

MelJuSo cells in 35 mm glass bottom dishes (MatTek) were incubated with 0.1 mg/ml of 70 kDa dextran conjugated with Texas Red (Molecular Probes) for 30 minutes at 37°C, while BMDCs were cultured on fibronectin-coated coverslips and incubated with 0.1 mg/ml of 70 kDa dextran conjugated with Texas Red for 15 minutes at 37°C. Cells were fixed in 3% PFA in PBS for 20 minutes at room temperature. Finally, cells were washed with PBS and distilled water and mounted with Mowiol (Sigma-Aldrich). Imaging was performed at the Olympus Fluoview FV1000 confocal laser scanning microscope equipped with a 60× PlanApo NA 1.35 objective.

For the analysis of macropinocytosis activity, the macropinocytic index (MI), corresponding to the percentage of cell area occupied by dextran-positive macropinosomes within a given time frame of dextran uptake (17) was calculated. First, the area of the cell was calculated (A_cell_) by drawing a region of interest (ROI) around each cell. Next, macropinosome area was obtained using particle analysis in ImageJ to identify dextran-positive objects. Prior to particle analysis, the images were processed with a Gaussian filter, and a threshold was applied to generate a binary image. The watershed function was used to split touching objects. Macropinosome area was added to obtain the total area of macropinosomes in the cell (A_t_). The macropinocytic index was calculated by dividing the total area of macropinosomes by the total area of the cell and multiplying it by 100 (MI = A_t_/A_cell_ * 100).

For the study of macropinosome closure, MelJuSo cells were transfected with PH-PLCδ-GFP 24 hours prior to the experiment. Cells were incubated with HLA-DR L243 Alexa Fluor 647 for 45 minutes and imaged every 30 seconds using a SoRa Spinning Disk microscope equipped with a UPLSAPO 60x/0.30 Silicon Oil objective. For the analysis of macropinocytosis in BMDCs migrating in microchannels, BMDCs were loaded into 5×8 μm microchannels (4D Cell). After 16 hours, channels were filled with 100 μg/ml 10 kDa Alexa Fluor 555-conjugated dextran (Invitrogen) for 50 minutes and then imaged every minute for 20 minutes using a SoRa Spinning Disk microscope equipped with a UPLSAPO 60x/0.30 Silicon Oil objective.

### Live cell imaging

Live-cell imaging was performed at 37°C with 5% CO_2_ on cells that were seeded on MatTek glass-bottom dishes. Live imaging of HLA-DR1 β-GFP stably transfected MelJuSo was performed using an inverted Olympus FluoView 1000 IX81 confocal laser scanning microscope equipped with a 60× PlanApo NA 1.35 objective. All the other live-cell imaging experiments, unless specified, were performed using the spinning disk Andor Dragonfly with a 60× Apo objective, NA 1.4.

### Image analysis, processing, and statistical analysis

Image processing and analysis were performed with FIJI (ImageJ) software (National Institute of Health). For the analysis of the MHC II uptake, the area of internalized MHC II was quantified by drawing a region of interest (ROI) around each cell and using particle analysis in ImageJ to identify MHC II-positive objects. Prior to particle analysis, the images were processed with a Gaussian filter, and a threshold was applied to generate a binary image. The watershed function was used to split touching objects.

For the analysis of the percentage of the HLA-DR1 β-GFP at the plasma membrane, HLA-DR1 β-GFP MelJuSo cells were selected with the freehand tool in FIJI to create selection 1. The Integrated Density of the GFP signal from the individual cells was measured to obtain the total amount of MHC II (IntDen1). The next step was to shrink selection 1 (Enlargement: -2) to create selection 2. The inside of selection 2 was cleared and the integrated density of selection 1 was measured again; this measurement corresponds to the signal of GFP at the plasma membrane (IntDen2). The percentage of HLA-DR1 β-GFP at the plasma membrane was obtained using the following formula: IntDen2/IntDen1*100.

Statistical analysis was performed using GraphPad Prism. Two-tailed unpaired Student’s *t*-tests were performed for the comparison of mean values between two groups from at least three independent experiments. Two-tailed paired Student’s *t*-tests were performed to compare the difference between a pair of means from at least three independent experiments in BMDCs. The p values (P) <0.05 were considered significant.

## Data availability statement

The original contributions presented in the study are included in the article/**Supplementary Material**. Further inquiries can be directed to the corresponding author.

## Author contributions

Conceptualization: NL, KZ, MM, PV, OB, and CP. Investigation: NL, KZ, MM, PV, and CP. Validation: NL and KZ. Formal analysis: NL and KZ. Methodology: NL, KZ, and PV. Funding acquisition: OB and CP. Project administration: CP. Writing—original draft preparation: NL and CP. Writing—review and editing: NL, KZ, MM, PV, OB, and CP. All authors contributed to the article and approved the submitted version.

## Supplementary material

Supplementary Figure 1 Rai14-GFP localizes to membrane ruffles and early-internalized vesicles. **(A)** Time-lapse video microscopy of MelJuSo cells transfected with Rai14-GFP. The arrows in the magnified boxes point to Rai14-positive membrane ruffles that are internalized into vesicles. Scale bar = 10 µm.

Supplementary Figure 2 Rai14 silencing retains MHC II at the plasma membrane. **(A)** FACS analysis of the surface and total expression levels of HLA-DR and Ii. Surface levels were measured in intact cells (left panels) and total levels were measured in saponin-permeabilized cells (right panels). The x-axis represents the mean fluorescence intensity of the conjugated markers indicated for each histogram. Shown are representative histogram overlays from one out of three independent experiments for control siRNA (blue) and Rai14 siRNA-treated cells (red). Control siRNA- and Rai14 siRNA-treated cells were also incubated with IgG2aK isotype control (dotted lines). **(B)** MelJuSo cells transfected with siRai14#1, siRai14#2, or siRNA control were incubated with an antibody against MHC II conjugated to Alexa Fluor 647 for 45 minutes and fixed at 0, 30, and 60 minutes after the treatment. The insets show magnifications of the boxed regions. Scale bar: 10 μm; inset: 2 μm.

Supplementary Figure 3 Rai14 silencing decreases myosin II recruitment at the front of migrating BMDCs. **(A)** BMDCs treated with siRNA control or siRai14 were loaded into 5×8 µm micro-fabricated channels, fixed after 16 h, and stained with an antibody against myosin II. The intensity of each cell for each condition was averaged into a single density map. The average from three independent experiments is shown. **(B)** Quantification of myosin II front-to-back ratio. Data represent the mean ± s.d. of three independent experiments (n>40 cells for each condition). *P<0.05 (two-tailed unpaired Student’s t-test).

Supplementary Video 1 Rai14-GFP localizes to membrane ruffles and early-internalized vesicles. MelJuSo cells transfected with Rai14-GFP were imaged with a Spinning Disk confocal microscope at 2-second intervals.

Supplementary Video 2 Rai14-GFP and Ii in live cells. An antibody targeting Ii (M-B741) conjugated with Alexa fluor 555 was added to MelJuSo cells co-transfected with Ii p33 and Rai14-GFP before live cell imaging. Cells were imaged with a Spinning Disk confocal microscope at 30-second intervals.

Supplementary Video 3 Rai14 and MHC II co-localize to membrane ruffles and forming macropinosomes. MelJuSo cells transfected with Rai14-GFP. Antibody targeting HLA-DR (L243) labeled with Alexa 647 was added to the culture medium immediately before imaging. The circle points to a Rai14 and MHC II-positive membrane ruffle that closes into a macropinosome. Cells were imaged with a Spinning Disk confocal microscope at 1-second intervals.

Supplementary Video 4 PtdIns(4,5)P_2_ is lost from the membrane of macropinosomes in MelJuSo cells treated with siRNA control. MelJuSo cells treated with siRNA control were transfected with PH-PLCδ-GFP to visualize PtdIns(4,5)P_2_. Antibody targeting HLA-DR (L243) labeled with Alexa 647 was added to the culture medium immediately before imaging. PtdIns(4,5)P_2_ and MHC II-positive membrane ruffle closes into a macropinosome and loses PtdIns(4,5)P_2_. Cells were imaged with a Spinning Disk confocal microscope at 30-second intervals.

Supplementary Video 5 PtdIns(4,5)P_2_ is retained at the membrane of macropinosome in MelJuSo cells silenced for Rai14. MelJuSo cells treated with siRai14 were transfected with PH-PLCδ-GFP to visualize PtdIns(4,5)P_2_. Antibody targeting HLA-DR (L243) labeled with Alexa 647 was added to the culture medium immediately before imaging. PtdIns(4,5)P_2_ and MHC II-positive membrane ruffle retains PtdIns(4,5)P_2_ at the macropinocytic cup. Cells were imaged with a Spinning Disk confocal microscope at 30-second intervals.
