# Supplementary figures and images for: Rai14 is a novel interactor of Invariant chain that regulates macropinocytosis
Source: Front Immunol. 2023 Jul 21;14:1182180. doi: 10.3389/fimmu.2023.1182180 (PMC10401043; doi:10.3389/fimmu.2023.1182180)

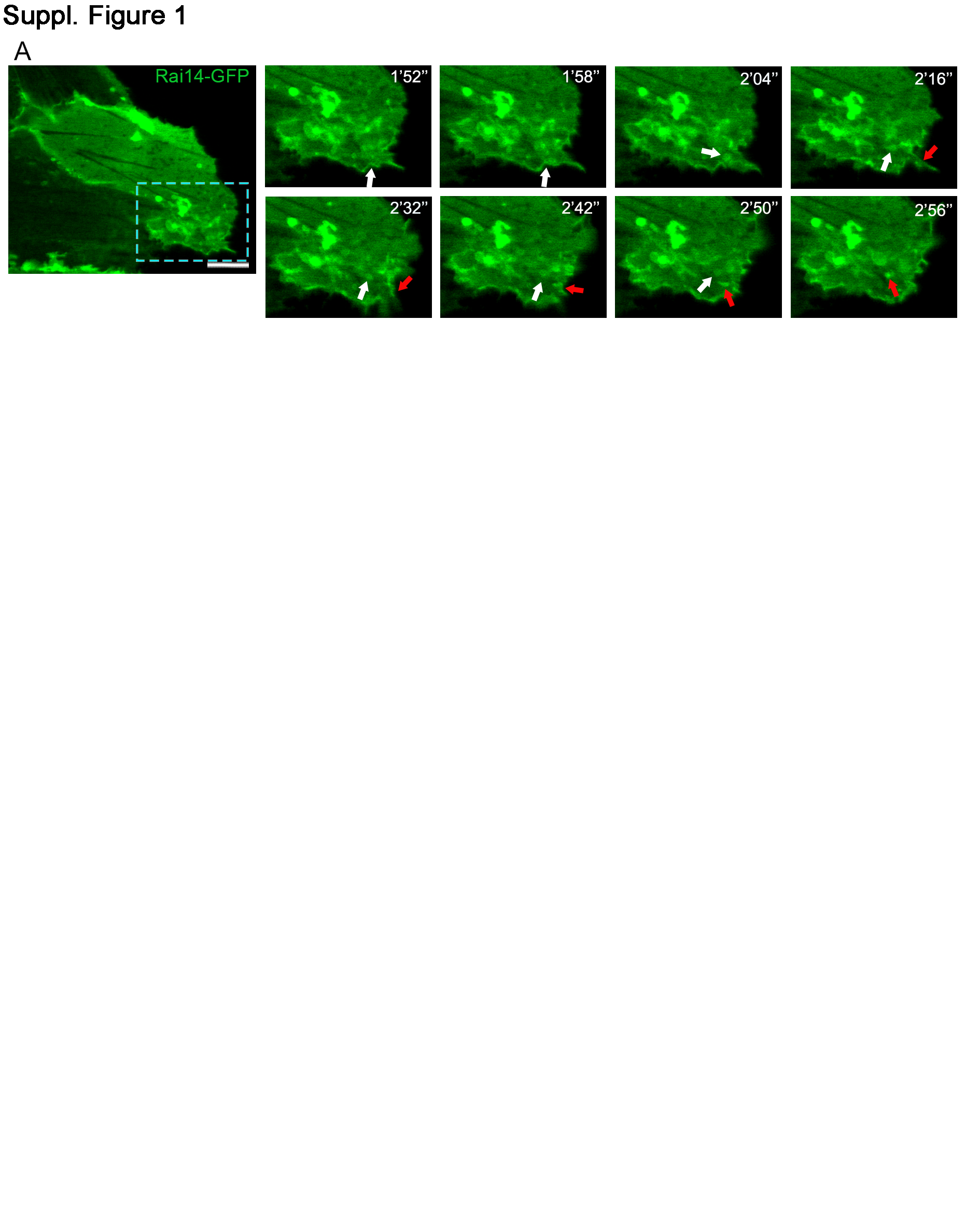

Supplement: Supplementary Figure 1 — Rai14-GFP localizes to membrane ruffles and early-internalized vesicles. (A) Time-lapse video microscopy of MelJuSo cells transfected with Rai14-GFP. The arrows in the magnified boxes point to Rai14-positive membrane ruffles that are internalized into vesicles. Scale bar = 10 µm. [file Image_1.tif]

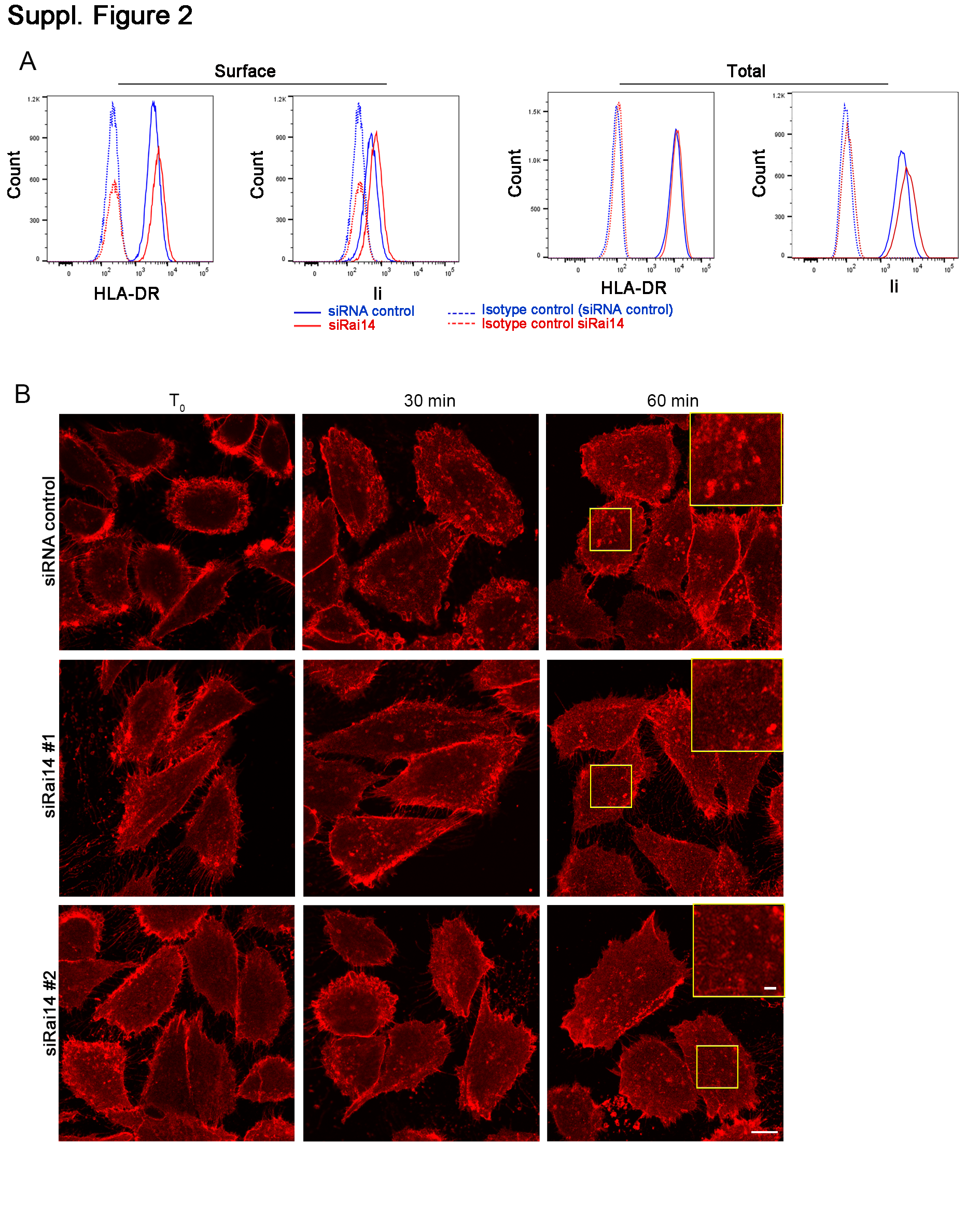

Supplement: Supplementary Figure 2 — Rai14 silencing retains MHC II at the plasma membrane. (A) FACS analysis of the surface and total expression levels of HLA-DR and Ii. Surface levels were measured in intact cells (left panels) and total levels were measured in saponin-permeabilized cells (right panels). The x-axis represents the mean fluorescence intensity of the conjugated markers indicated for each histogram. Shown are representative histogram overlays from one out of three independent experiments for control siRNA (blue) and Rai14 siRNA-treated cells (red). Control siRNA- and Rai14 siRNA-treated cells were also incubated with IgG2aK isotype control (dotted lines). (B) MelJuSo cells transfected with siRai14#1, siRai14#2, or siRNA control were incubated with an antibody against MHC II conjugated to Alexa Fluor 647 for 45 minutes and fixed at 0, 30, and 60 minutes after the treatment. The insets show magnifications of the boxed regions. Scale bar: 10 μm; inset: 2 μm. [file Image_2.tif]

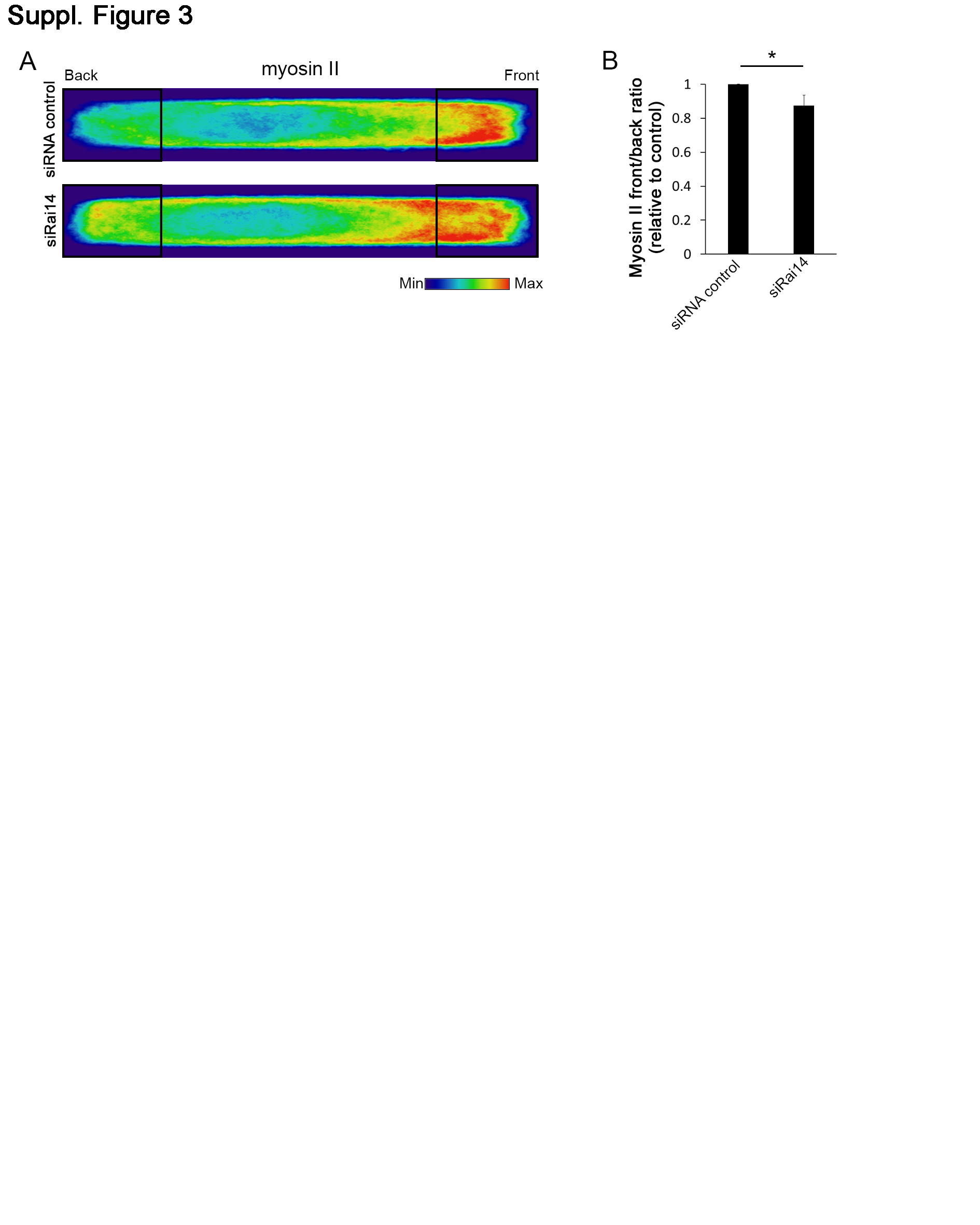

Supplement: Supplementary Figure 3 — Rai14 silencing decreases myosin II recruitment at the front of migrating BMDCs. (A) BMDCs treated with siRNA control or siRai14 were loaded into 5×8 µm micro-fabricated channels, fixed after 16 h, and stained with an antibody against myosin II. The intensity of each cell for each condition was averaged into a single density map. The average from three independent experiments is shown. (B) Quantification of myosin II front-to-back ratio. Data represent the mean ± s.d. of three independent experiments (n>40 cells for each condition). *P<0.05 (two-tailed unpaired Student’s t-test). [file Image_3.tif]
